# Toll-Like Receptor 4 Deficiency Accelerates the Development of Insulin-Deficient Diabetes in Non-Obese Diabetic Mice

**DOI:** 10.1371/journal.pone.0075385

**Published:** 2013-09-23

**Authors:** Elke Gülden, Masaru Ihira, Atsushi Ohashi, Anna Lena Reinbeck, Marina A. Freudenberg, Hubert Kolb, Volker Burkart

**Affiliations:** 1 Institute for Clinical Diabetology, German Diabetes Center, Leibniz Center for Diabetes Research, Member of the German Center for Diabetes Research (DZD), Düsseldorf, Germany; 2 Department of Developmental Immunology, Max-Planck-Institute of Immunbiology and Epigenetics, Freiburg, Germany; University of British Columbia, Canada

## Abstract

**Background/Objective:**

Toll-like receptors (TLR) mediate the recognition of microbial constituents and stress-induced endogenous ligands by the immune system. They may also be involved in the maintenance or break down of tolerance against autologous antigens. The aim of our investigation was to study the consequence of TLR4 deficiency on the development of insulin-deficient diabetes in the NOD mouse.

**Methods:**

The TLR4 defect of the C57BL/10ScN mouse was backcrossed onto the NOD background and the effect of TLR4 deficiency on diabetes development was analysed by in vivo and in vitro studies.

**Results:**

Compared to animals with wildtype TLR4 expression (TLR4^+/+^), female NOD mice carrying a homozygous TLR4 defect (TLR4^−/−^), showed significant acceleration of diabetes development, with a younger age at diabetes onset (TLR4***^+/+^*** 177±22 d, TLR^−/−^: 118±21 d; *p*<0.01). Pancreata of 120 d old TLR4^−/−^ NOD mice revealed increased proportions of islets with advanced stages of immune cell infiltration compared to TLR4^+/+^ mice (*p*<0.05). TLR4 deficiency did not affect the susceptibility of islet cells to the beta cell damaging mediators nitric oxide or the inflammatory cytokines tumor necrosis factor alpha, interleukin-1 beta and interferon gamma. The lack of TLR4 further had no effect on the frequency of regulatory T-cells but reduced their capacity to inhibit T-cell proliferation.

**Conclusions:**

Our findings demonstrate that TLR4 deficiency results in an acceleration of diabetes development and immune cell infiltration of islets in NOD mice. We conclude that TLR4 is involved in the progression of the insulitis process thereby controlling the development of insulin-deficient diabetes in NOD mice.

## Introduction

Mammalian toll-like receptors (TLRs) comprise a family of phylogenetically conserved transmembrane proteins that play a central role in the elicitation of immune responses against microbial pathogens [1]. TLR ligands comprise viral, bacterial as well as fungal structures and TLR signalling is important for the induction of innate as well as adaptive immunological host defence mechanisms. Due to these properties TLRs are considered important links between the innate and the adaptive immune response [2]. Recent investigations provide evidence that TLR functions are not strictly limited to the recognition of exogenous, microbial antigens. TLRs were found to interact also with endogenous (host-derived) ligands [3] such as mammalian stress proteins [4], [5], degradation products of hyaluronic acid [6], beta defensin [7] and DNA fragments including (hypomethylated) CpG motifs [8]. The capacity to regulate innate and adaptive immune reactivity combined with the ability to recognize exogenous as well as autologous antigen ligands qualify TLRs for central roles not only in host resistance against microbial pathogens but also in the control of immunological tolerance and in the development of autoimmune disorders [9]. In fact, accumulating evidence points to an involvement of TLR-dependent processes in the pathogenesis of systemic and organ-specific autoimmune disorders, such as rheumatoid arthritis [10], multiple sclerosis [11] and systemic lupus erythematosus [12] and in the development of contact allergy [13], [14].

Type 1 diabetes is a severe metabolic disease characterised by absolute insulin deficiency as the consequence of the immune-mediated destruction of autologous insulin-producing pancreatic beta cells [15]. Although the pathogenic processes involved in the initiation and progression of the disease are not completely understood, numerous studies in patients with type 1 diabetes and in animal models of the human disease demonstrate that the beta cell-directed proinflammatory state is induced by innate and adaptive immunity [16], [17].

TLR4 represents the central component of the mammalian receptor complex for lipopolysaccharide (LPS), a constituent of the outer cell wall of gram-negative bacteria. This qualifies TLR4 as a critical structure in host defence against bacterial infections [18]. However, TLR4 was also found to provide efficient protection against inflammatory tissue damage by promoting repair and tissue remodelling processes as demonstrated in lung and gut injury [19], [20]. Further studies revealed that TLR4 is also able to bind endogenous structures, including heat shock protein 60 (Hsp60) [4], a dominant stress protein and a putative beta cell autoantigen assumed to be involved in the pathogenesis of type 1 diabetes [21]. In mammals, the expression of TLR4 was initially described on cells of the innate immune system, predominantly macrophages [22], which were found to contribute to the initiation and progression of beta cell-directed immune reactivity [23]. Recent investigations further proved the expression of TLR4 also by regulatory T-lymphocytes (Treg) which are characterised by the coexpression of the surface structures CD4 and CD25 [24]. Treg cells protect the host by limiting the magnitude of immune responses and by preventing the activation of immunological effector mechanisms directed against autologous structures [25]. Insufficient Treg activity may result in reduced self-tolerance and the promotion of autoimmune reactivity. Studies on the potential role of Treg in the development of type 1 diabetes in fact point to an association of the disease with defective Treg activity [26].

In order to study the possible role of TLR4 in diabetes development, we generated a TLR4-deficient strain of the NOD mouse, the currently best characterized model of human type 1 diabetes [27] and analysed diabetes progression by in vivo and in vitro approaches.

## Materials and Methods

### Animals

NOD mice were from the breeding colony at the German Diabetes Center. C57BL/10ScN mice lacking TLR4 expression due to a spontaneous deletion of the TLR4-encoding region on chromosome 4 [28], [29], but carrying a functional IL-12 receptor β2 chain [30], were from the Max-Planck-Institute of Immunbiology and Epigenetics, Freiburg, Germany. To transfer the TLR4 defect allele onto the NOD background, male C57BL/10ScN mice (TLR4^−/−^) were bred with female NOD mice (TLR4^+/+^) and the TLR4 heterozygous male offspring (TLR4^+/−^) were then backcrossed for more than 12 generations with female NOD mice to preserve NOD specific mitochondrial DNA. Heterozygous littermates from backcross generations 12–15 were intercrossed to generate TLR4^+/+^, TLR4^+/−^ and TLR4^−/−^ animals at proportions that followed a Mendelian distribution of 1∶2∶1. PCR analyses from genomic DNA were performed to confirm successful backcrossing of the TLR4 defect and to prove homozygosity of the NOD specific alleles of the diabetes-associated loci Idd 1, 2, 3 and 15 as described previously [29], [31]. The sequences of the primers used for PCR analyses and the lengths of the resulting products are listed in Table 1. The animals had free access to water and food. Comparable proportions of female TLR4^+/+^, TLR4^+/−^ and TLR4^−/−^ mice were monitored for the development of diabetes until 220 d of age. Animals were considered diabetic with blood glucose levels (measured on an EPOS Analyzer 5060, Eppendorf, Hamburg, Germany) exceeding 14 mmol/l on two consecutive days. Islet cells and immune cell populations were isolated from normoglycemic female mice.

**Table 1:** Sequences of oligonucleotides used for PCR analyses of TLR4 expression status and NOD-specific alleles of diabetes-associated loci.

			Product length	
Gene/gene locus	Forward primer (5′–3′)	Backward primer (5′–3′)	C57BL/10	NOD
TLR4 wildtype	CAG TCG GTC AGC AAA CGC CTT CTT	CAA GGC AGG CTA GCA GGA AAG GGT G	401	401
TLR4 defect	GCA AGT TTC TAT ATG CAT TCT C	CCT CCA TTT CCA ATA GGT AG	140	140
β-actin	GGC CCA GAG CAA GAG AGG TA	GGT TGG CCT TAG GGT TCA GG	176	176
IDD1 (D17Mit34)	TGT TGG AGC TGA ATA CAC GC	GGT CCT TGT TTA TTC CCA GTA CC	148	126
IDD2 (D9Mit25)	AAA CCC AGT CTT AAA AAC AAA ACA	TTC ATT TTA TTT TCT TTG GAA AGG	130	136
IDD3 (D3Mit95)	CTA AAA GCA CTA GCA AAG AAA ATC A	CCT CCA CAC ACA TGT CCT TG	122	146
IDD15 (D5Mit48)	GAC TAT CAT CCA AGC CAA GAC C	AAA AGA CAC TTT CCC TGA CAT AGC	206	150

### Ethics Statement

The experiments were approved by the ethics committee on animal welfare of the State of North Rhine Westphalia and conducted in accordance with the Principles of Laboratory Animal Care.

### Macrophage Enrichment and Stimulation

Single cell suspensions were prepared from spleens and seeded on FCS (Gibco-BRL, Life Technologies, Rockville, CA) coated petri dishes. After 2 h of incubation (37°C, 5% CO_2_), the non-adherent cells were removed and the adherent, macrophage-enriched cell fraction was detached. The cells were seeded in 96 well microtiter plates (2×10^5^ cells per well) in medium RPMI 1640 (PAA Laboratories, Linz, Austria) supplemented with 10% FCS, ampicillin (25 mg/l), penicillin (120 mg/l), streptomycin (270 mg/l), 1 mM sodium pyruvate, 2 mM glutamine, non essential amino acids (10 ml/l, 100×), 24 mM NaHCO_3_ and 10 mM HEPES (culture medium). The cells were stimulated with lipopolysaccharide (LPS, from *Escherichia coli* EH100, Enzo Life Sciences, Lörrach, Germany) or macrophage-activating lipopeptide 2 (MALP-2, kindly provided by P. Mühlradt, Braunschweig, Germany) [32]. After incubation (37°C, 5% CO_2_) for 6 and 24 h, respectively, the concentrations of tumor necrosis factor α (TNFα) and interleukin-6 (IL-6) in the culture supernatants were quantified by ELISA (OptEIA, BD Biosciences, Heidelberg, Germany).

### Pancreas Histology

After paraffin-embedding, from each pancreas serial thin sections (5 µm) from at least three levels separated by about 500 µm were stained with hematoxylin. For insulin staining, anti-porcine insulin serum from guinea pig (Dako, Hamburg, Germany) was used as primary antibody and the Vectastain ABC Kit with anti-guinea pig IgG (Camon, Wiesbaden, Germany) as detection system. The percentage of the islet area occupied by inflammatory cells was determined morphometrically using an interactive microscopical image analysis system (Axiovision, Carl Zeiss, Göttingen, Germany). From each pancreas 28.1±4.1 areas of the stained sections were evaluated.

### Isolation of Pancreatic Islet Cells and Cytotoxicity Assay

Pancreatic islets of normoglycemic 70-day-old female mice were isolated by collagenase digestion of pancreatic tissue and dispersed into single cells by trypsin treatment as described [33]. Due to the very low number of islets/islet cells that could be isolated from (prediabetic) animals, appropriate experimental procedures were selected based on the results of pilot studies, our previous results and on reports from the literature [34]–[36]. In brief, islet cells were seeded in 96 well microtiter plates (1×10^4^ cells per well) and incubated (37°C, 5% CO_2_) in the absence or presence of mixtures of the recombinant murine cytokines TNFα, interleukin-1 beta (IL-1β) and interferon γ (IFNγ) (R&D Systems, Wiesbaden, Germany) for 6 d or of the nitric oxide (NO)-donor diethylenetriamine-NO (DETA-NO) (Situs-Chemicals, Düsseldorf, Germany) for 24 h. At the end of the incubation periods, the percentage of dead cells was determined by analysing adherent as well as non-adherent cells using the trypan blue exclusion assay as described [34], [37].

### Isolation and Functional Characterization of CD4^+^CD25^+^ Treg Cells

Single cell suspension of spleens from TLR4^+/+^ and TLR4^−/−^ mice were tested for their proliferative activity by BrdU-incorporation (2×10^5^ cells per well of a 96 well microtiter plate) according to the manufacturer’s instruction (Roche Applied Science, Mannheim, Germany). The functional characterisation of Treg cells focused on spleen-derived CD4^+^CD25^+^ cells as the spleen was the only organ that allowed isolation of CD4^+^CD25^+^ Treg and CD4^+^CD25^−^ Tresponder cells at sufficient quantity and purity to perform the experiments. CD4^+^CD25^+^ cells were enriched from spleen cell suspensions by a magnetic bead separation technique (Miltenyi Biotec, Bergisch Gladbach, Germany). To confirm the purity, cells were stained with a FITC-conjugated monoclonal rat anti-mouse CD4 antibody (BD Pharmingen, Heidelberg, Germany) and with a phycoerythrin (PE)-conjugated monoclonal rat anti-mouse CD25 antibody (BD Pharmingen). The expression of Foxp3 was assessed by staining of permeabilized cells with a PE-conjugated monoclonal rat anti-mouse Foxp3 antibody (eBioscience, San Diego, CA, USA) in combination with an anti-CD4 antibody. The samples were analyzed in a FACSCalibur flow cytometer (BD Biosciences).

To investigate the LPS-responsiveness of the Treg populations, CD4^+^CD25^+^ cells from TLR4^+/+^ and TLR4^−/−^ mice were seeded in 96 well microtiter plates (1×10^4^ cells per well) and exposed to increasing LPS concentrations. After 6 d the metabolic activity of the cells was determined by their ability to reduce the water soluble tetrazolium salt WST-1 (Roche Applied Science). The concentration of the resulting formazan salt was determined photometrically at 420 nm (reference wavelength 600 nm).

The inhibitory capacity of the Treg populations was assessed in a coculture assay [38], [39]. CD4^+^CD25^+^ Treg and CD4^+^CD25^−^ responder cells were coincubated at different ratios in the wells of 96 well microtiter plates precoated with hamster anti-mouse CD3 antibody (0.5 µg/ml, BD Pharmingen). After 5 d of cultivation in the presence of 0.1 ng/ml recombinant murine interleukin-2 (IL-2, 4 U/ng, R&D Systems, Wiesbaden), the metabolic activity of the cocultures was determined by the WST-1 assay.

### Statistical Analysis

Data were expressed as mean values+SD and statistical analysis was performed using the Student’s *t* test. To analyse the kinetics of diabetes development, data were plotted in Kaplan-Meier survival curves and data sets were compared by application of the logrank test. Differences were considered statistically significant with *p*<0.05. All statistical analyses were performed using the Prism software package version 4 (GraphPad Software, San Diego, CA, USA).

## Results

### Accelerated Development of Spontaneous Diabetes in TLR4-deficient Female NOD Mice

**Figure 1:**
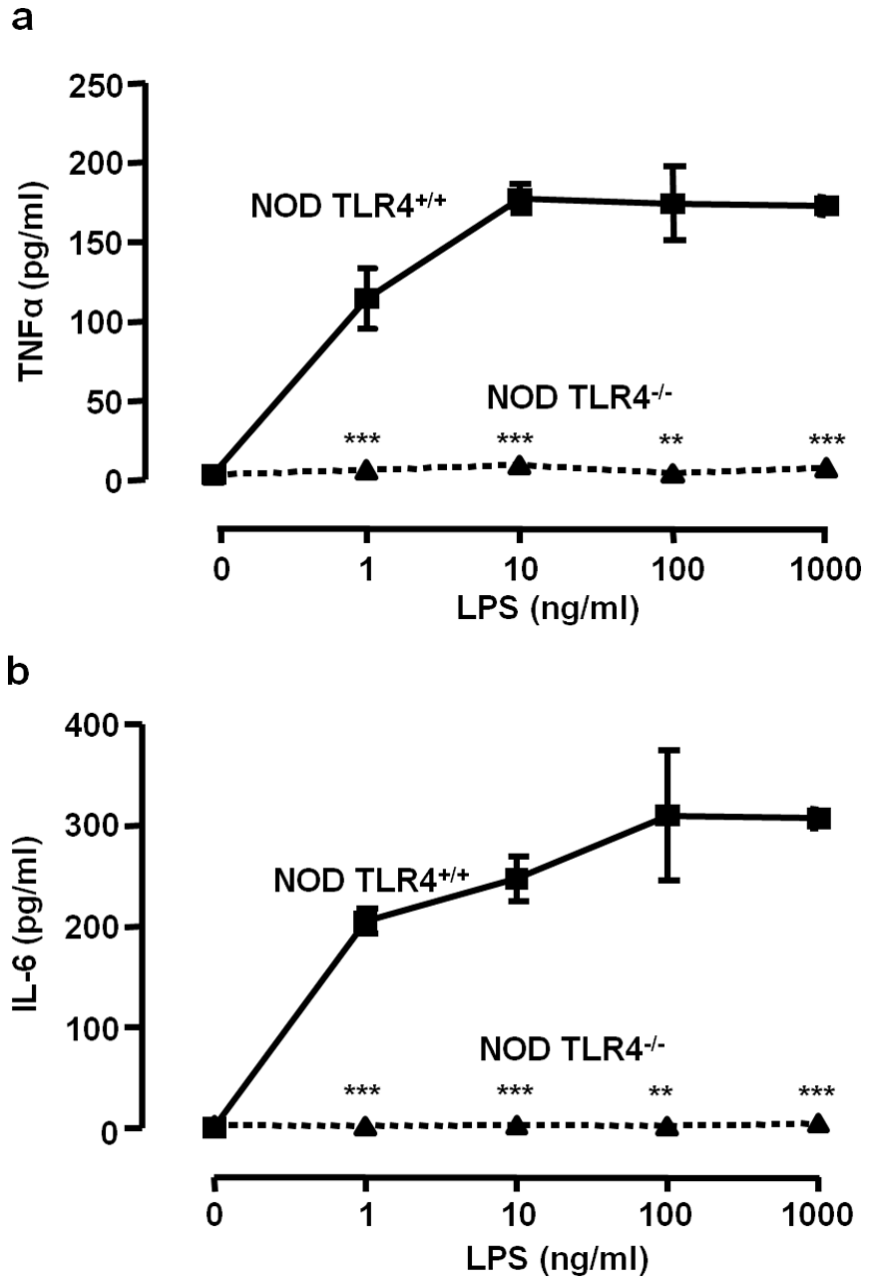
Deficient LPS-responsiveness in macrophage-enriched spleen cell fractions of TLR4^−/−^ NOD mice. Macrophage-enriched spleen cell fractions of TLR4^+/+^ (squares) and TLR4^−/−^ (triangles) NOD mice were cultivated in the presence of increasing concentrations of LPS (0–1000 ng/ml) (**a, b**) or, in addition, with MALP-2 (100 ng/ml) (**c**). After an incubation of 6 h and 24 h, respectively, the concentrations of accumulated TNFα and IL-6 were determined in the culture supernatants by ELISA. The data show means ± SD from determinations performed in triplicates. **p<0.01; ***p<0.001 compared to the corresponding data of TLR4^+/+^ cells.

TLR4^+/+^ and TLR4^−/−^ NOD mice derived from backcross generations 12–15 were tested for reactivity to the TLR4 ligand LPS. Macrophage-enriched spleen cell populations were exposed to highly purified LPS and the accumulation of TNFα and IL-6 in the culture supernatants were determined by ELISA after 6 and 24 h, respectively. As shown in Figure 1, cell populations from TLR4^+/+^ mice responded to LPS challenge with the accumulation of high concentrations of up to 177.4±9.3 pg/ml TNFα (10 ng/ml LPS, Figure 1a) and of up to 310.0±64.4 pg/ml IL-6 (100 ng/ml LPS, Figure 1b), whereas cells of TLR4^−/−^ mice did not release elevated TNFα or IL-6 levels, even after exposure to 1000 ng/ml LPS. The TLR2 ligand MALP-2 [32] induced comparable concentrations of TNFα in populations of TLR4^+/+^ (12.3±4.2 pg/ml) and TLR4^−/−^ mice (10.1±2.4 pg/ml) (Figure 1c).

**Figure 2:**
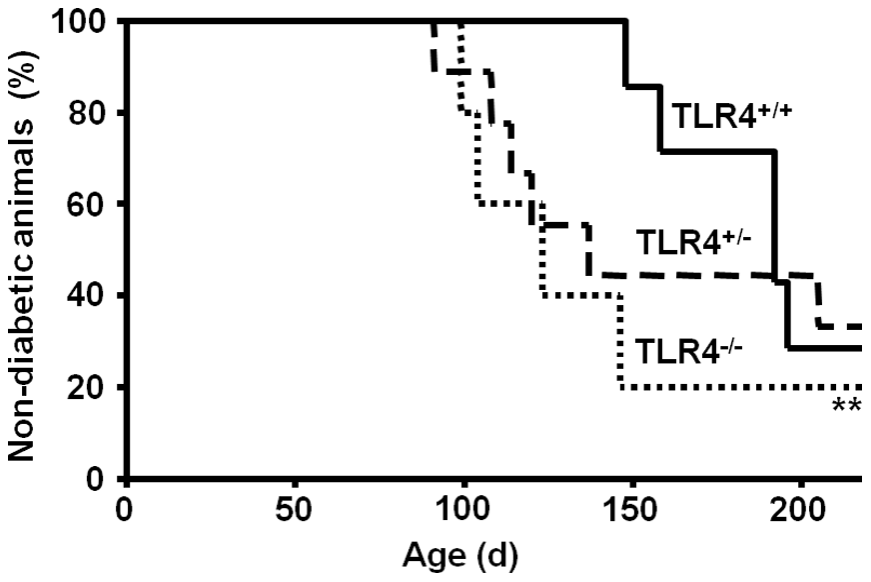
Acceleration of spontaneous diabetes development in TLR4-deficient female NOD mice. The development of diabetes was monitored by following the same proportions of female mice with homozygous TLR4 expression (TLR4^+/+^, solid line) and with heterozygous (TLR4^+/−^, dashed line) or homozygous TLR4 defect (TLR4^−/−^, dotted line) generated from intercrosses and backcrosses of C57BL/10ScN and NOD mice. The data show the development of spontaneous diabetes as the percentage of non-diabetic animals until an age of 220 d. Of each genotype 10–12 animals (littermates) were observed over the same period of time. **p<0.01 compared to the kinetics of diabetes development in TLR4^+/+^ or TLR4^+/−^ NOD mice.

The effect of TLR4 deficiency on the development of spontaneous diabetes in NOD mice was assessed by monitoring disease manifestation (BG>14 mmol/l on two consecutive days) in female TLR4^+/+^, TLR4^+/−^ and TLR4^−/−^ mice until an age of 220 d. In the group of TLR4^+/+^ mice the first case of diabetes was observed at an age of 148 d, and until 210 d of age 71% of the animals had developed diabetes (mean age of diabetes manifestation 177±22 d) (Figure 2). These kinetics of diabetes development largely correspond to that observed in the parental NOD mouse strain maintained at the German Diabetes Center [31]. Mice with homozygous or heterozygous TLR4 deficiency exhibited a significant acceleration of diabetes development. TLR4^−/−^ mice showed a mean age of diabetes manifestation of only 118±21 d (*p*<0.01 compared to TLR4^+/+^ mice), representing an acceleration by 59 d. In TLR4^+/−^ mice the mean age of diabetes manifestation was 129±40 d (*p*<0.01 compared to TLR4^+/+^ mice), corresponding to an acceleration by 48 d. Total diabetes rates were not significantly different between the groups (TLR4^+/+^: 71%, TLR4^+/−^: 67%, TLR4^−/−^: 80%).

### Enhanced Infiltration of Pancreatic Islets in TLR4-deficient mice

**Figure 3:**
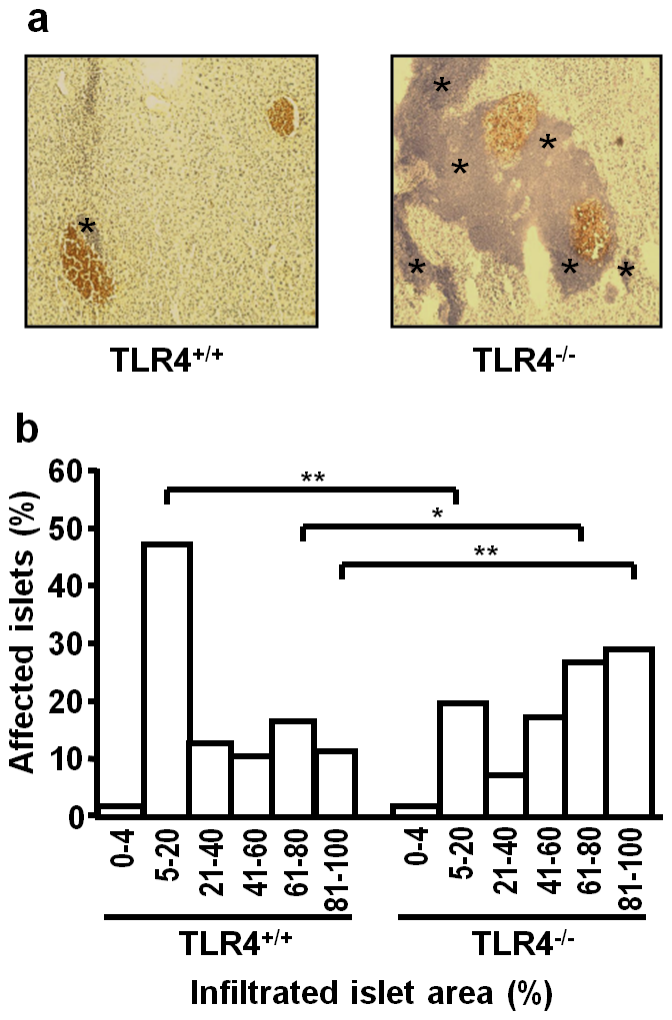
Severe immune cell infiltration in the pancreas of TLR4-deficient NOD mice. Thin sections of pancreatic tissue from 120^+/+^ and TLR4^−/−^ mice were stained for insulin and counterstained with hematoxylin. The micrographs show representative pancreatic tissue sections with areas of insulin containing beta cells stained in brown and regions infiltrated by immune cells (blue areas, asterisks) (**a**). The degree of islet infiltration was assessed morphometrically by determining the islet area occupied by infiltrating immune cells as percent of the whole islet area (**b**). The numbers of islets with a certain degree of infiltration from all examined animals of each genotype (TLR4^+/+^ and TLR4^−/−^) were added and expressed as cumulative proportions of afflicted islets. The number of all investigated islets of each genotype was set 100%. Per genotype 140–220 islets from 4–5 animals were evaluated. *p<0.05; **p<0.01.

Histological analyses of pancreata from normoglycemic mice at the age of 120 d revealed increased proportions of islets with pronounced mononuclear infiltration and advanced stages of insulitis in TLR4^−/−^ animals when compared to TLR4^+/+^ animals (Figure 3). Moreover, in TLR4^−/−^ mice, infiltration frequently remained not restricted to the islet area but extended from the islet periphery into the surrounding exocrine tissue (Figure 3a). Morphometric analyses revealed initial stages of immune cell infiltration (5–20% of the islet area affected) in 47.0% of the islets of TLR4^+/+^ mice, but in only 20.5% of the islets of TLR4^−/−^ mice (*p*<0.01) (Figure 3b). In contrast, islets with severe immune cell infiltration (>80% of the islet area affected) were observed more frequently in TLR4^−/−^ mice (28.4%) than in TLR4^+/+^ mice (11.4%) (*p*<0.01). To detect a potential effect of the expanding inflammation on the absolute size of the residual beta cell area in islets of TLR4^−/−^ mice, the insulin positive area was determined in (i) islets with <5% inflamed area, (ii) islets with inflammation remaining restricted to the islet area, and (iii) islets with inflammation extending into the surrounding tissue. Morphometric analyses of 138 islets revealed insulin positive areas of 21842±3623 µm^2^ (mean ± SD) in islets with <5% infiltration (i), 28014±5752 µm^2^ in islets with intra-insulitis (ii) and 27881±9380 µm^2^ in islets with inflammation extending into the surrounding tissue (iii). No significant differences could be observed between the groups, indicating that particularly the excessive form of inflammation (iii) does not affect the size of the residual insulin positive beta cell area in islets of normoglycemic, prediabetic TLR4^−/−^ NOD mice.

### No Impact of TLR4 Deficiency on Islet Cell Susceptibility to Cytotoxic Stress

**Figure 4:**
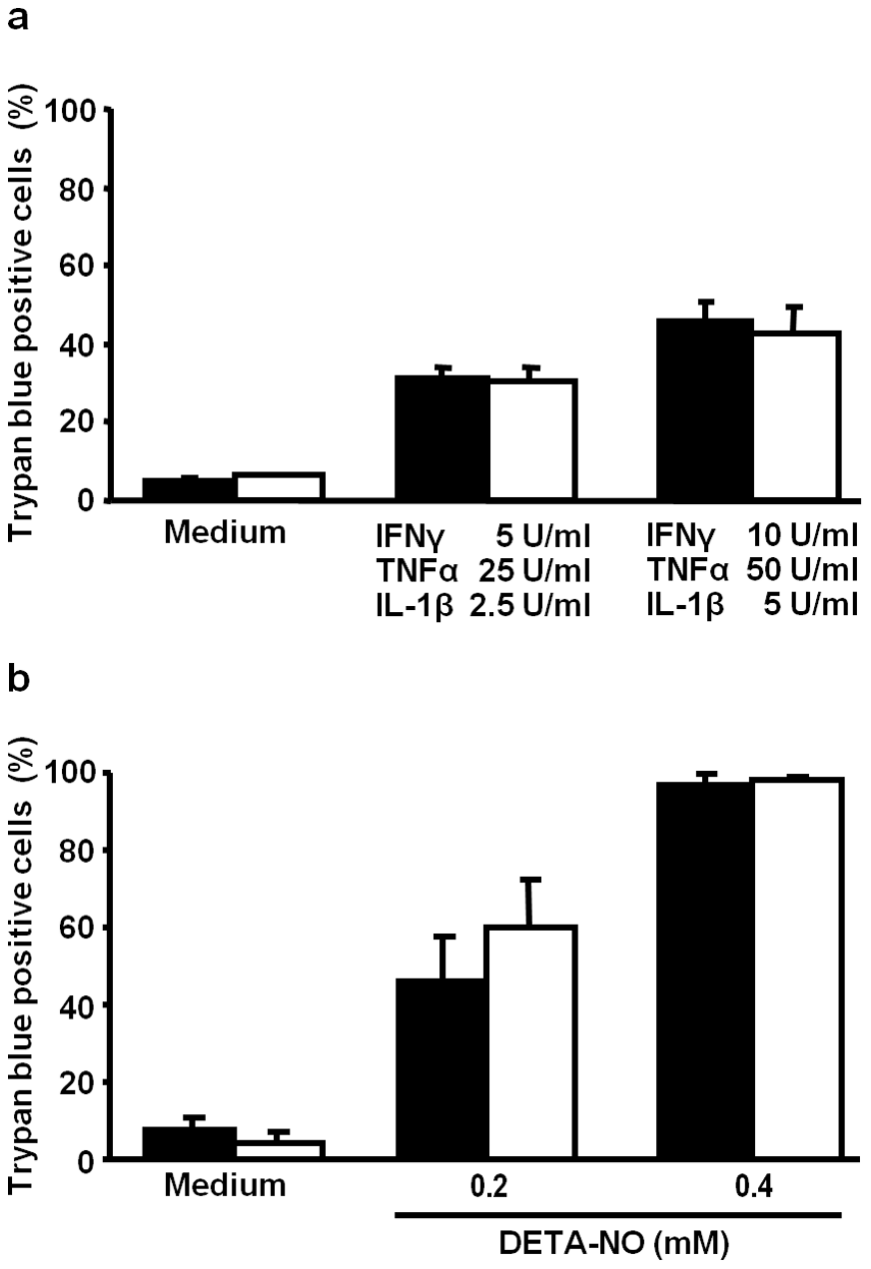
Islet cell susceptibility to beta cell-damaging mediators is not affected by the TLR4 expression status. Islet cells of TLR4^+/+^ (solid bars) and TLR4^−/−^ mice (open bars) were cultivated in the absence (Medium) or in the presence of a mixture of the inflammatory cytokines IFNγ, TNFα and IL-1β (6 d) (**a**) or the NO-donor DETA-NO (24 h) (**b**). At the end of the incubation period the proportion of dead cells was determined by the trypan blue exclusion assay. The data show means+SD from three experiments performed in triplicates.

To investigate whether accelerated diabetes development and enhanced islet infiltration in TLR4-deficient NOD mice is caused by an increased susceptibility of their pancreatic beta cells towards inflammatory mediators we exposed cultivated islet cells of TLR4^+/+^ and TLR4^−/−^ mice to a mixture of TNFα, IL-1β and IFNγ or to the NO-donor DETA-NO (Figure 4). After 6 d of cytokine exposure 46±5% of TLR4^+/+^ and 42±7% of the TLR4^−/−^ islet cells were dead, as quantified by the trypan blue exclusion assay (Figure 4a). DETA-NO at a concentration of 0.2 mM induced the death of 46±11% of TLR4^+/+^ and 60±12% of TLR4^−/−^ islet cells, whereas 0.4 mM of the NO-donor induced the death of >95% of both islet cell populations (Figure 4b).

### Effects of TLR4 Deficiency on IL-2-dependent Proliferation and Treg Activity

**Figure 5:**
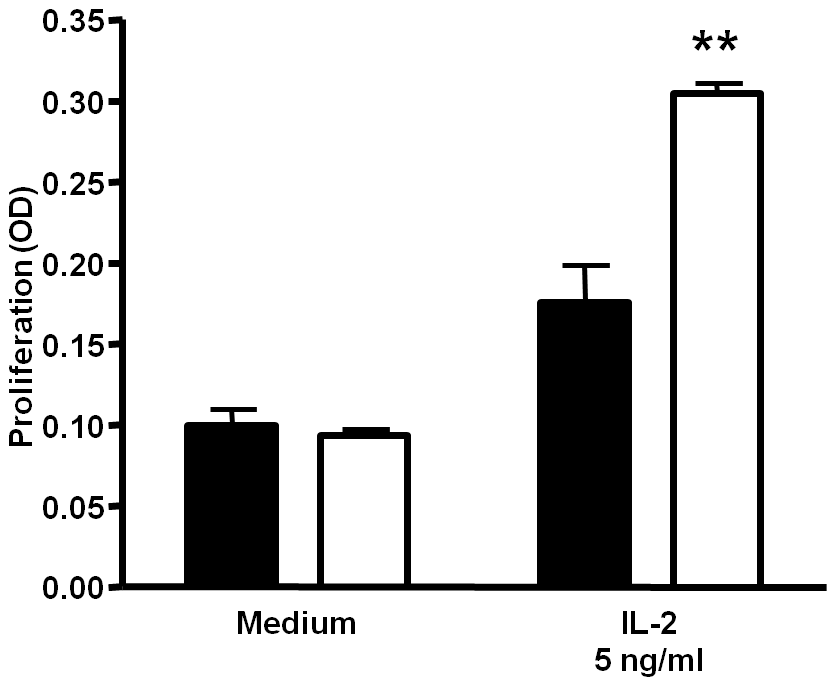
Increased proliferative response in spleen cells of TLR4^−/−^ mice. Spleen cells from 85–100 d old TLR4^+/+^ (solid bars) and TLR4^−/−^ mice (open bars) were incubated for 72 h in the absence (medium) or presence of 5 ng/ml IL-2. The proliferative activity of the cells was determined by BrdU-incorporation. The data show means+SD from three experiments performed in triplicates. **p<0.01 compared to TLR4^+/+^ cells.

As a first approach to assess a potential effect of TLR4 expression on T-cell reactivity in NOD mice, we determined the proliferation of isolated spleen cells in response to IL-2. As shown in Figure 5, there was a significantly increased proliferative response to IL-2 of spleen cells from TLR4^−/−^ mice when compared to splenocytes from TLR4^+/+^ animals.

**Figure 6:**
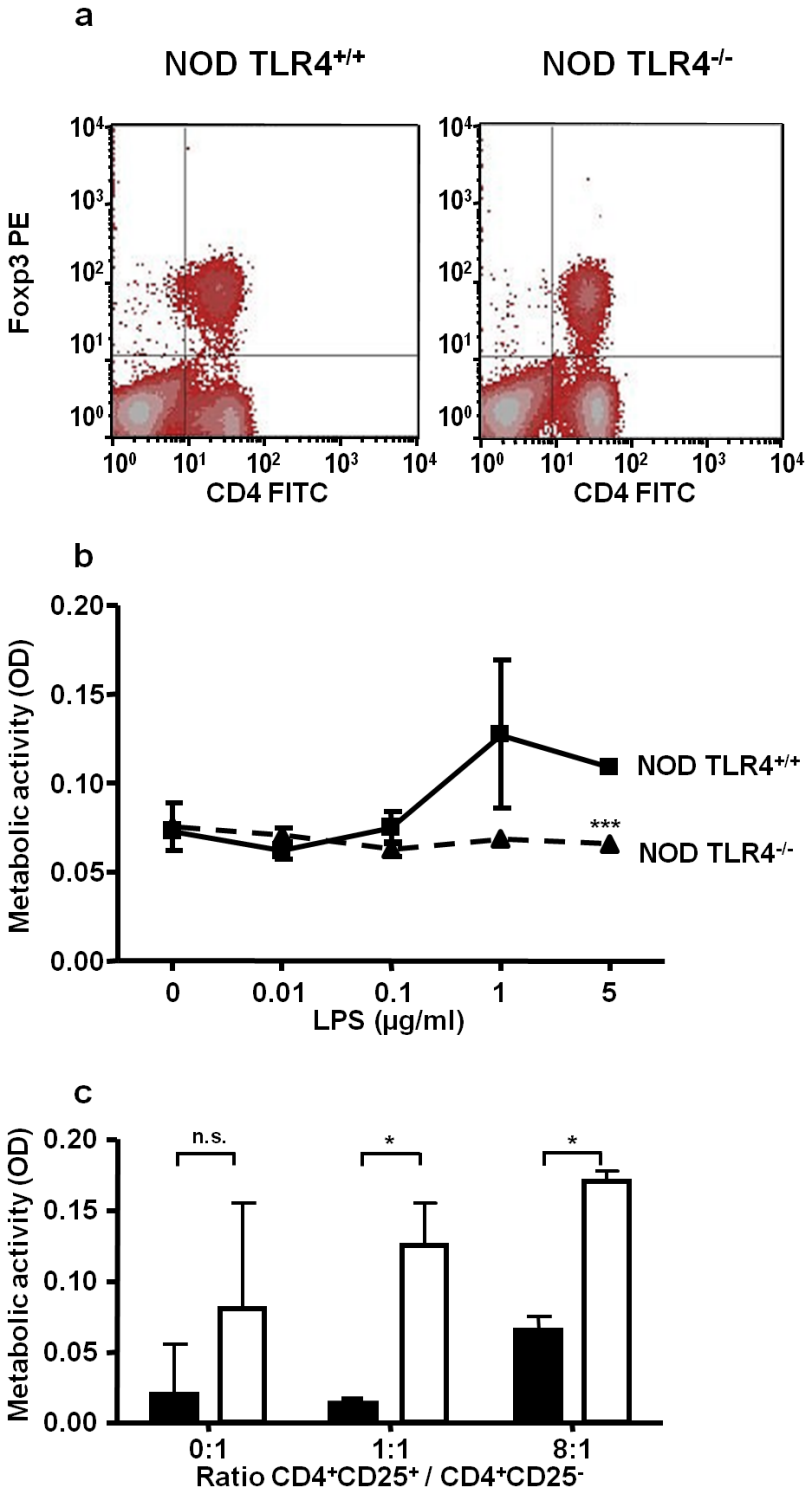
The TLR4 expression status does not affect Treg frequency but Treg activity in NOD mice. Unseparated spleen cells suspensions from female TLR4^+/+^ and TLR4^−/−^ NOD mice were stained with FITC-conjugated anti-CD4 antibodies and PE-conjugated anti-Foxp3 antibodies. The resulting signals were quantified by FACS analysis. The diagrams show the results of a representative FACS analysis (**a**). CD4^+^CD25^+^ Treg cells isolated from TLR4^+/+^ (squares) and TLR4^−/−^ NOD mice (triangles) were exposed to increasing concentrations of LPS (**b**). After 6 d of culture the metabolic activity of the cells was determined by their capacity to convert the tetrazolium salt WST-1 into its water-soluble formazan product. The data show means ± SD from three experiments performed in triplicates. **p<0.01 compared to the TLR4^−/−^ cells. (**c**) To assess the inhibitory capacity of Treg, CD4^+^CD25^+^ Treg cells and CD4^+^CD25^−^ responder cells were isolated from the spleens of TLR4^+/+^ (solid bars) and TLR4^−/−^ NOD mice (open bars) by magnetic bead separation. Treg cells and responder cells were cocultivated at various ratios for 5 d in the presence of anti-CD3 antibodies and IL-2. The metabolic activity of the cocultures was assessed by their WST-1 conversion capacity. *p<0.05 compared to the cocultures of the TLR4^+/+^ cells; n.s. not significant.

Since the primary T-cell subpopulation responsible for restricting T-cell proliferation comprises Treg cells, the possible effect of TLR4 deficiency on Treg frequency was analysed by determining the proportion of CD4^+^Foxp3^+^ cells in suspensions of total spleen cells from TLR4^+/+^ and TLR4^−/−^ mice. As shown in Figure 6a, density blots generated from FACS analyses of permeabilized spleen cells revealed almost identical proportions of CD4^+^Foxp3^+^ cells in animals of both genotypes indicating that the TLR4 expression status does not affect Treg frequency in NOD mice.

To analyse the functional activity of Treg cells, they were isolated from spleen cell suspensions of TLR4^+/+^ and TLR4^−/−^ NOD mice by a magnetic bead separation technique that permits the enrichment of viable and functionally active CD4^+^CD25^+^ Treg cells by avoiding the use of permeabilising agents. FACS analyses of the resulting cell fractions con-firmed that the separation procedure yields highly purified CD4^+^CD25^+^ Treg populations (>96% purity) from TLR4^+/+^ as well as TLR4^−/−^ mice (not shown). Incubation of purified CD4^+^CD25^+^ cells from TLR4^+/+^ mice in the presence of increasing LPS concentrations resulted in an increase of the metabolic activity of the cells (Figure 6b). In contrast, cells from TLR4^−/−^ mice were unresponsive to the bacterial stress signal.

To assess the effect of the TLR4 expression status on the inhibitory potential of Treg cells from NOD mice, we incubated purified CD4^+^CD25^−^ responder cells in the absence or presence of various ratios of CD4^+^CD25^+^ Treg cells. After five days of incubation in the presence of anti-CD3 antibodies and IL-2 we determined the metabolic activity of the CD4^+^ T-cell populations as a measure of their activation status. When CD4^+^CD25^−^ responder cells were incubated in the absence of Treg cells, both responder cell types showed a large variation in metabolic activity, but no significant difference between TLR4^+/+^ and TLR4^−/−^ cells was observed (Fig. 6c). As expected, cultivation of CD4^+^CD25^−^ cells in the presence of increasing numbers of CD4^+^CD25^+^ Treg cells led to an increase of the metabolic activity of TLR4^+/+^ as well as TLR4^−/−^ cell populations due to increased total cell numbers in the assay samples. However, the activity of TLR4^−/−^ mouse-derived cell populations was consistently higher than in the cell populations isolated from TLR4^+/+^ animals. Even in the presence of an eight-fold excess of Treg cells, the activity of TLR4^−/−^ cells remained significantly higher than in TLR4^+/+^ cell populations. This observation points to an impaired suppressive activity in the CD4^+^ T-cell population of TLR4^−/−^ NOD mice.

## Discussion

To test the hypothesis that TLR4 activity is involved in the pathogenesis of insulin-deficient diabetes, we established a NOD mouse line that selectively lacks the expression of TLR4. The model was generated by backcrossing animals of the NOD mouse strain with mice of the C57BL/10ScN strain that carries a spontaneous deletion of the entire TLR4 encoding region [29], [30]. For a functional proof of the successful transfer of the TLR4 defect onto the NOD background we exposed macrophage-enriched spleen cell populations to the TLR4 ligand LPS. This induced the release of substantial amounts of TNFα and IL-6 from the cell population of TLR4 expressing NOD mice. In contrast, cells from TLR4-deficient mice were completely unresponsive to LPS. The lack of TLR4 expression did not significantly affect the responsiveness of the cell populations to the TLR2 ligand MALP-2 [40].

Monitoring diabetes manifestation in TLR4-deficient NOD mice revealed an accelerated onset of overt diabetes in female animals with heterozygous and homozygous TLR4 deficiency by a mean of 59 and 48 d, respectively. The finding of an earlier disease onset in TLR4-deficient animals implicates a role for TLR4 as a regulator of the pathomechanisms involved in diabetes development. Indeed, there was a strong accelerating effect of TLR4 deficiency on the insulitis process. Histological examinations of pancreatic tissue of normoglycemic mice at the age of 120 d revealed predominant patterns of peri-insular monocytic/lymphocytic infiltration in islets of female NOD mice with wildtype TLR4 expression but advanced stages of intra-insular infiltration in islets of TLR4-deficient animals (NOD TLR4^−/−^ and NOD TLR4^+/−^). Whereas in NOD TLR4^+/+^ mice inflammation remained strictly limited to the islet area, the pancreatic tissue of TLR4-deficient animals (NOD TLR4^−/−^ and NOD TLR4^+/−^) shows a vast expansion of the inflamed area into the islet-surrounding tissue, apparently without affecting the size of the residual beta cell area. The spreading of inflammatory reactivity from its site of origin to initially unaffected, healthy tissue is observed in various disease states including microbial infections, inflammatory bowel disease, malignancies and autoimmunity [41]–[44].

Since TLR4 is expressed by human and mouse pancreatic beta cells [45], [46] it may impact the disease process at this level. Engagement of TLR4 on beta cells by LPS leads to a decrease in insulin content and secretion [46] which is caused, at least in part, by LPS-induced mediators, such as CXCL10 [47]. Signalling of the chemokine CXCL10 via TLR4 also impairs beta cell function [47]. Therefore, it is an interesting observation that although a direct activation of insulin-producing beta cells via TLR4 impairs their function, the absence of TLR4 results in an acceleration of diabetes development. To address this issue, we tested whether the absence of TLR4 on beta cells modulates their susceptibility towards toxic immune mediators. Hence, we exposed islet cells isolated from TLR4-competent and TLR4-deficient NOD mice to NO or a mixture of inflammatory cytokines which had previously been identified as potent beta cell damaging mediators [48]. We observed a highly similar, dose-dependent cell death response in cultivated islet cells irrespective of the TLR4 expression status of their donors. This finding largely rules out the assumption that increased susceptibility of TLR4-deficient beta cells to autoaggressive immune mechanisms contributes to accelerated disease progression in NOD mice lacking TLR4 expression.

Alternatively, TLR4 deficiency may promote disease development by modulating the immune system. In animal models of type 1 diabetes we and others provided evidence that the initiation of pancreatic islet inflammation critically depends on functionally active macrophages or other antigen presenting cells [23], [49]. The lack of TLR4 on innate immune cells such as dendritic cells or macrophages may limit the proinflammatory or immunostimulatory capacity of these cells rather than promote immune reactivity. Indeed, TLR4 deficiency of antigen presenting cells appears to impair the experimental induction of cellular immunity in mouse models [50]. However, in our current study, the presence of islet inflammation in both, TLR4^+/+^ and TLR4^−/−^ mice, demonstrates that the insulitis-triggering capacity of antigen presenting cells in NOD mice is not impaired by TLR4 deficiency. The finding of enhanced inflammation in TLR4-deficient animals rather points to a dysregulation of later stages of islet inflammation. An enhanced insulitis process as observed in TLR4^−/−^ animals may therefore be due to a dysfunction of the regulatory T-cell population which controls the critical balance between the stimulation of immune reactivity to ensure efficient host protection and the activation of counterregulatory mechanisms to prevent damage of normal healthy tissue by an overreacting immune system [25].

This functional property qualifies Treg cells as key regulators in the control of (auto-) immune reactivity. Recent findings demonstrate that the TLR expression status has profound effects on the Treg cell population. When compared to wild type mice, animals with a selective TLR2 defect exhibit a decreased frequency of Treg cells in their visceral fat depot [51] with a potential impact on the inflammatory processes in adipose tissue and on systemic (subclinical) inflammation. Moreover, the capacity of Treg cells to restrain immune reactivity was found to depend on the expression of functionally active TLR4 [24]. We therefore hypothesised that TLR4-deficient NOD mice, showing accelerated diabetes development, exhibit decreased Treg cell activity. Since the inhibitory potential of a Treg population is determined by its number and/or functional activity, we tested for both alternatives in our animal model.

FACS analyses revealed that the status of TLR4 expression of the mice does not affect the number or proportion of their Treg cells. However, in TLR4-deficient mice these cells could no more be activated by a TLR4 agonist, such as occurring naturally by endogenous heat shock proteins [52]. More detailed, functional analyses revealed that the capacity to inhibit the activation of the CD4^+^CD25^−^ responder T-cell population was significantly reduced in CD4^+^CD25^+^ Treg cells from TLR4-deficient mice when compared to Treg cells from TLR4-expressing animals. These findings fit with the result of a meta-analysis indicating that the occurrence of type 1 diabetes is not associated with a decreased number or proportion of Treg cells but with a functional impairment of this cell population as defined by its decreased suppressive capacity [53].

Interestingly, NOD mice lacking the adaptor molecule MyD88 involved in intracellular TLR signalling, were protected from diabetes when kept under normal specific pathogen-free conditions whereas germ-free mutants still developed diabetes [54]. This suggests that the gut microbiota mediates protection from diabetes in the absence of MyD88 but not in its presence. Some TLRs are able to deliver signals also in the absence of MyD88. In particular, MyD88 independent signalling has been found for TLR3 and TLR4 [55]. This fits with the heterogenous outcome of TLR defects in NOD mice. While TLR2 and TLR9 knockout mice show little development of diabetes, a defect of TLR3 appears to be without impact [56], [57] and, as we show here, deficiency in TLR4 causes enhancement of the disease process. Whether the protective action of TLR4 is mediated by ligands from the gut, such as LPS, or from other tissues, such as heat shock proteins, remains to be determined. Indeed, repeated administration of TLR4 agonists (LPS) have been reported previously to attenuate the disease process in NOD mice [58], [59]. Although secondary effects of these treatment regimens, such as LPS-mediated (cyto-)toxicity and/or tolerisation, cannot be excluded, the outcome of these experiments also point to a role of TLR4 in the development of diabetes in the NOD mouse.

Two other studies have mentioned the generation of NOD TLR4-deficient mice, in one case the type of TLR4 deficiency is not mentioned [54], in the other case the TLR4 mutant gene from the C3H/HeJ mouse was introduced onto the NOD background [56]. Both studies report that TLR4-deficient NOD mice develop diabetes but it was not described whether there was acceleration of diabetes development compared to the parental NOD mouse strain. However, in our current study, by introducing the selective TLR4 defect of the C57BL/10ScN mouse onto the NOD mouse background we were able to provide conclusive evidence for the involvement of TLR4 in the progression of insulin-deficient diabetes.

Taken together, our results demonstrate that the progression of insulin-deficient diabetes in NOD mice is under control of TLR4. Such a regulatory function has also been observed in animal models of other organ-specific autoimmune disorders like encephalomyelitis [60]. Further detailed studies are required to identify the critical TLR4-dependent step(s) in the disease process as the basis for the development of TLR4-directed intervention strategies.
